# Synthetic Cinnamides and Cinnamates: Antimicrobial Activity, Mechanism of Action, and In Silico Study

**DOI:** 10.3390/molecules28041918

**Published:** 2023-02-17

**Authors:** Mayara Castro de Morais, Edeltrudes de Oliveira Lima, Yunierkis Perez-Castillo, Damião Pergentino de Sousa

**Affiliations:** 1 Department of Pharmaceutical Sciences, Federal University of Paraíba, João Pessoa 58051-900, PB, Brazil; 2 Bio-Cheminformatics Research Group and Area de Ciencias Aplicadas, Facultad de Ingeniería y Ciencias Aplicadas, Universidad de Las Americas, Quito 170503, Ecuador; 3 Postgraduate Program in Bioactive Natural and Synthetic Products, Federal University of Paraíba, João Pessoa 58051-900, PB, Brazil

**Keywords:** cinnamic acid, natural product, medicinal plant, esters, amides, molecular docking, mechanism of action, antimicrobial, *S. aureus*, *Candida*

## Abstract

The severity of infectious diseases associated with the resistance of microorganisms to drugs highlights the importance of investigating bioactive compounds with antimicrobial potential. Therefore, nineteen synthetic cinnamides and cinnamates having a cinnamoyl nucleus were prepared and submitted for the evaluation of antimicrobial activity against pathogenic fungi and bacteria in this study. To determine the minimum inhibitory concentration (MIC) of the compounds, possible mechanisms of antifungal action, and synergistic effects, microdilution testing in broth was used. The structures of the synthesized products were characterized with FTIR spectroscopy, ^1^ H-NMR, ^13^ C-NMR, and HRMS. Derivative **6** presented the best antifungal profile, suggesting that the presence of the butyl substituent potentiates its biological response (MIC = 626.62 μM), followed by compound **4** (672.83 μM) and compound **3** (726.36 μM). All three compounds were fungicidal, with MFC/MIC ≤ 4. For mechanism of action, compounds **4** and **6** directly interacted with the ergosterol present in the fungal plasmatic membrane and with the cell wall. Compound **18** presented the best antibacterial profile (MIC = 458.15 μM), followed by compound **9** (550.96 μM) and compound **6** (626.62 μM), which suggested that the presence of an isopropyl group is important for antibacterial activity. The compounds were bactericidal, with MBC/MIC ≤ 4. Association tests were performed using the Checkerboard method to evaluate potential synergistic effects with nystatin (fungi) and amoxicillin (bacteria). Derivatives **6** and **18** presented additive effects. Molecular docking simulations suggested that the most likely targets of compound **6** in *C. albicans* were caHOS2 and caRPD3, while the most likely target of compound **18** in *S. aureus* was saFABH. Our results suggest that these compounds could be used as prototypes to obtain new antimicrobial drugs.

## 1. Introduction

**Figure 1:**
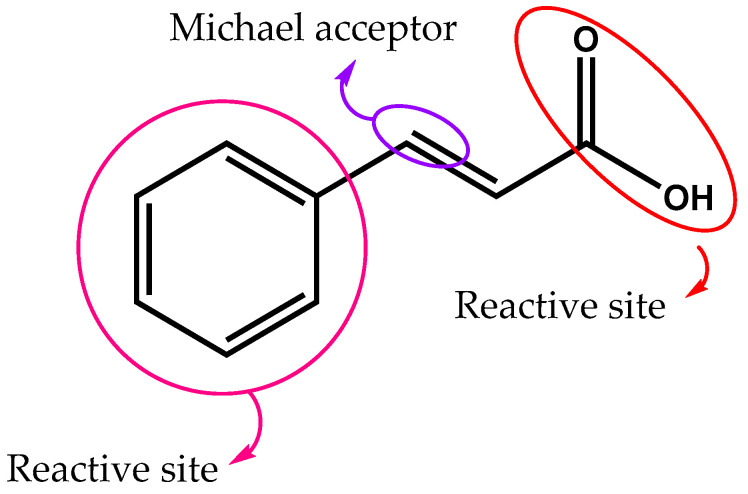
Reactive sites in the CA structure.

Cinnamic acid (CA) (**1**) is a natural product that presents a low toxicity to most living organisms. It is found in plant species, mainly in *Cinnamomum zeylanicum*, which gives rise to the term “cinnamic”. Its structure is simple (Figure 1), but it is involved in many crucial systemic functions such as growth, development, reproduction, and defense in various plant species [1,2]. This acid comes from the biosynthetic pathway of shikimic acid, its precursor being phenylalanine, and participates in the synthesis of more complex secondary metabolites such as lignins, flavonoids, isoflavonoids, tannins, coumarins and anthocyanins, which play vital roles in plant physiology such as during growth, development, reproduction and disease resistance [3,4].

In the last 10 years, interest in CA and its derivatives (esters, amides, aldehydes and alcohols) [5] has increased because of its many pharmacological activities, such as antituberculosis [6,7,8,9], antiviral [10], anticancer [11,12,13,14,15,16], mammary (MCF-7) and prostate (PC-3) inhibition, neoplastic cell growth, apoptosis inducement [17,18,19], anticholesterolemic [20,21,22,23], cardioprotective [24], antioxidant [25,26,27,28], anti-inflammatory [2,29,30,31,32], hepatoprotective [33,34,35], antidiabetic [36,37,38], antimalarial [39], anxiolytic [40], central nervous system depressant [41], neuroprotective [42,43,44,45,46], larvicidal agent [47,48,49,50,51], anti-leishmania [52], cosmetic [17,53], and antimicrobial action [54,55,56,57,58,59,60,61,62,63,64,65,66] properties.

Infectious diseases caused by bacteria and fungi remain a major global health problem, especially in developing countries. Antibacterial and antifungal drugs, widely known as antimicrobials, began to be used in chemotherapy in the 1940s, and many new classes of antimicrobials were discovered in the period from 1940 to 1970 and successfully introduced into clinical practice [67]. However, as these new drugs were employed, drug-resistant strains began to appear [68]. The widespread use of antibiotics during the last forty years in animal feed and human consumption has only accelerated the emergence of resistance in various pathogenic organisms [69]. Infection with drug-resistant strains is typically associated with longer treatments, greater toxicity, and higher costs.

The CA skeleton is an interesting scaffold for the development of novel bioactive substances. CA derivatives are known for their antimicrobial activity [21]. Antimicrobial mechanism studies have reported that CA mainly exerts its activity through plasma membrane disruption, nucleic acid and protein damage, and the induction of intracellular reactive oxygen species [70,71]. The biological activities of CA can be explained through its interactions with the molecular targets present in a living organism. CA has three main reactive sites: the aromatic ring substituent, the carboxylic acid function, and the conjugated olefin (Figure 1) [72].

Therefore, the objectives of this study were to synthesize a series of cinnamic acid derivatives and to evaluate their antifungal and antibacterial activities to identify new antimicrobial drug candidates. An in silico approach was used to investigate the potential mechanisms of antimicrobial action.

## 2. Results and Discussion

### 2.1. Chemistry

**Figure 2:**
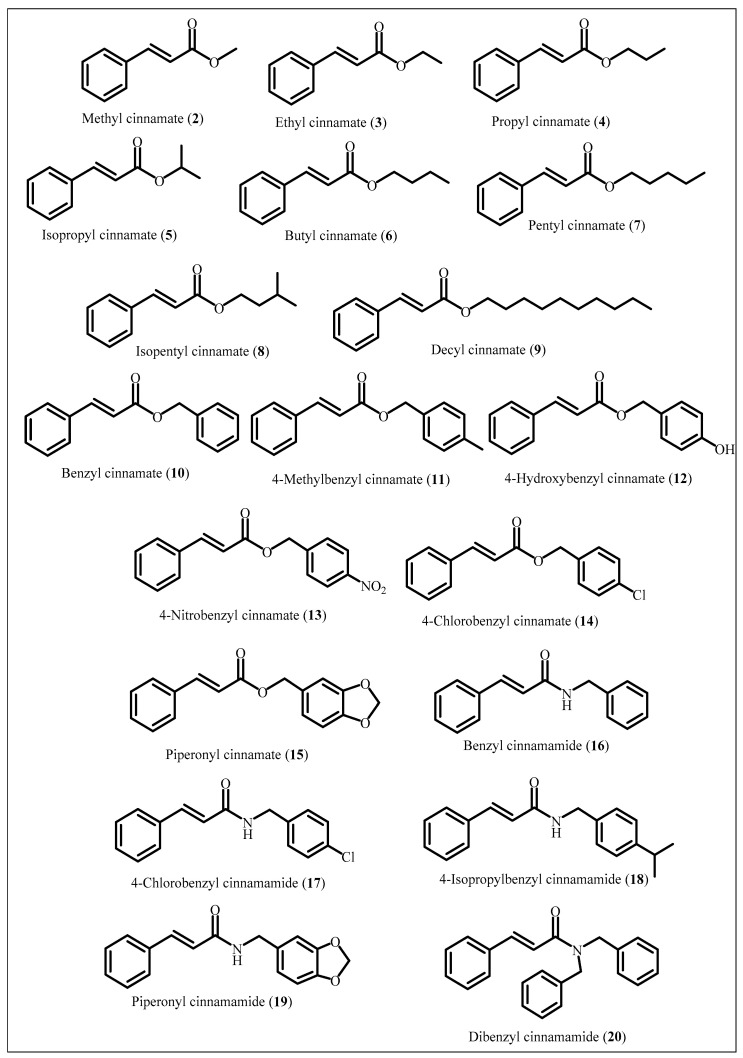
Chemical structures of the prepared compounds.

**Scheme 1:**
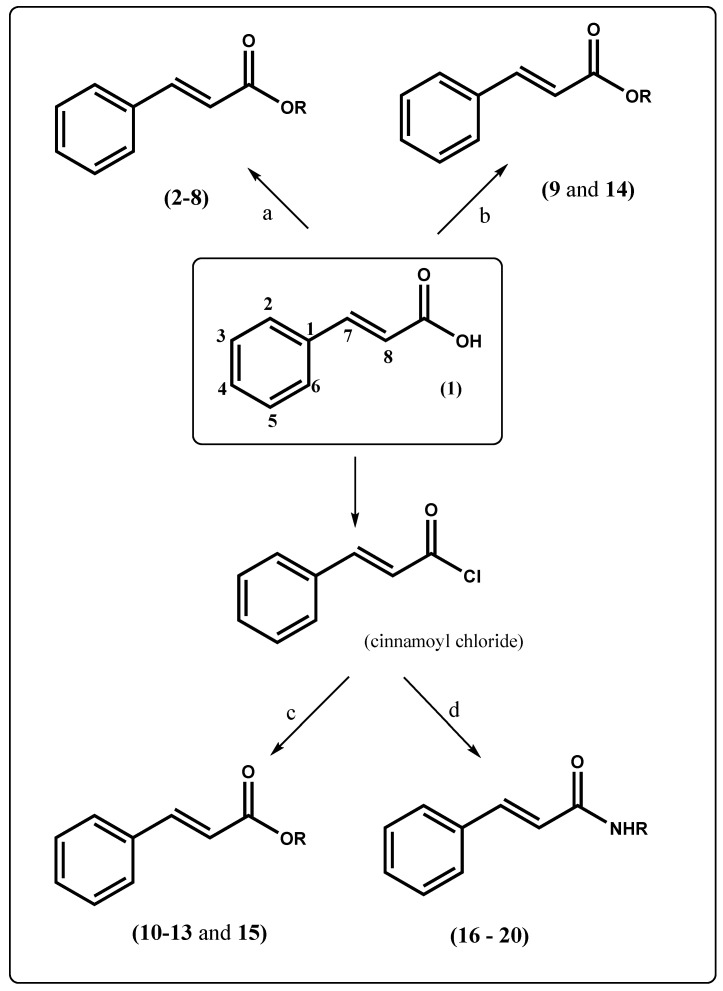
Synthesis of cinnamic acid derivatives: (**a**) ROH, H_2_SO_4_, reflux; (**b**) Et_3_N, RX, acetone, reflux; (**c**) ROH, pyridine, reflux; (**d**) RNH_2_, pyridine, reflux.

Cinnamic acid (**1**) and cinnamoyl chloride were used as starting materials for the preparation of a series of nineteen compounds: via Fischer esterification reactions [51,73,74] (**2**–**8**), bimolecular nucleophilic substitution [51,73,75] (**9** and **14**), and Schotten–Baumann reactions [65] (**10**–**13** and **15**–**20**) (Scheme 1). The following reagents were used: methanol (**2**), ethanol (**3**), propanol (**4**), isopropanol (**5**), butanol (**6**), pentanol (**7**), iso-pentanol (**8**), bromodecane (**9**), benzyl alcohol (**10**), 4-methylbenzyl alcohol (**11**), 4-hydroxybenzyl alcohol (**12**), 4-nitrobenzyl alcohol (**13**), 4-chlorobenzyl chloride (**14**), piperonyl alcohol (**15**), benzyl amine (**16**), 4-chlorobenzyl amine (**17**), 4-isopropylbenzyl amine (**18**), piperonyl amine (**19**) and dibenzyl amine (**20**) (Figure 2). The compounds were structurally characterized using infrared (IR) spectroscopy, nuclear magnetic resonance (NMR), and high-resolution mass spectrometry.

The compounds were evaluated for in vitro antimicrobial activity against strains of *Candida albicans* (ATCC-76485), *Candida tropicalis* (ATCC-13803), *Candida glabrata* (ATCC-90030), *Aspergillus flavus* (LM-171), *Penicillium citrinum* (ATCC-4001), *Streptococcus aureus* (ATCC-35903), *Streptococcus epidermidis* (ATCC-12228), and *Pseudomonas aeruginosa* (ATCC-25853).

An analysis of the ^1^ H NMR spectra of the cinnamic derivatives showed seven hydrogens in common in the cinnamoyl substructure, five belonging to the aromatic ring and two olefinic hydrogens referring to the side carbon chain. In view of the common signals for all analogues, the most unprotected hydrogen signal in the spectrum was that of the olefinic hydrogen in the form of a doublet at about δ_H_ 7.60 ppm, which was coupled to the neighboring hydrogen that presented a signal in the form of doublet around δ_H_ 6.53 ppm, evidencing the configuration of the double bond is *trans.*

In the ^13^ C NMR spectra, chemical shifts indicated that the cinnamic derivatives had nine carbons in common. A signal close to δ_C_ 166.0 ppm was attributed to carbonyl; a signal around δ_C_ 141.3 ppm was attributed to olefinic carbon. A signal at δ_C_ 133.0 ppm was attributed to the aromatic carbon adjacent to the olefinic group. In addition, the signals at about δ_C_ 129.6 ppm and δ_C_ 128.8 ppm were attributed to *meta* carbons of the aromatic ring and to two ortho carbons, respectively, and another chemical shift of approximately δ_C_ 127.8 ppm indicated carbon in the *para* position. Furthermore, the presence of a signal close to δ_C_ 120.3 ppm was attributed to olefinic carbon.

The IR spectra showed absorption bands between 2850 and 3000 cm^−1^, indicating C-H sp3 stretching; signals between 3000 and 3100 cm^−1^ indicating C-H sp2 stretching; and C=O stretching bands in the range of 1750–1730 cm^−1^ characteristic of ester carbonyls or a strong signal around 1650 cm^−1^ belonging to the carbonyl stretch of the amides.

### 2.2. Evaluation of Antifungal Activity

Following the antifungal activity analysis of the nineteen cinnamic acid analogs against strains of *C. albicans* (ATCC-76485), *C. tropicalis* (ATCC-13803), *C. glabrata* (ATCC-90030), *A. flavus* (LM-171), and *P. citrinum* (ATCC-4001) (Table 1), it was verified that eleven were bioactive. Butyl cinnamate (**6**) was the most potent compound (MIC = 626.62 µM) against all tested strains. Ethyl cinnamate (**3**) and methyl cinnamate (**2**) were also bioactive, though with a lower potency, with MIC = 726.36 µM and 789.19 µM, respectively. In general, the ester derivatives were more bioactive than the amide derivatives.

#### 2.2.1. Minimum Fungicidal Concentration (MFC)

One may analyze whether a substance is fungicidal or fungistatic by calculating its MFC to MIC ratio [76]. Therefore, MFC/MIC ratios were calculated to determine whether the substances presented fungistatic (MFC/MIC > 4) or fungicidal (MFC/MIC ≤ 4) activity. The MFC/MIC ratios were all less than 4 (Table 2), strongly suggesting that the compounds maintain antifungal activity with a fungicidal character.

#### 2.2.2. Mechanism of Action

Sterols make up part of the constitution of all fungal cells. Ergosterol is a principal sterol and modulates membrane fluidity, cell growth, and proliferation [76]. In this study, tests to detect the biological targets of compounds **4** and **6** were performed by adding ergosterol to the medium. An increase in the MIC of the compounds was observed, indicating that the fungus likely acts by inhibiting ergosterol synthesis or directly binding to ergosterol. Azoles and polyenes are well-known classes of antifungal drugs that act on ergosterol to treat fungal infections [76] (Table 3 and Table 4).

When studying cell wall architecture, protoplasts stabilized with osmo-protectants are important biochemical tools [76]. Osmotic stability is used with *C. albicans* and other fungi to study antibiotic mechanisms of action [76,77,78]. Damage to essential components of the cell wall, caused by antifungal agents such as cell wall synthesis inhibitors, can lyse cells in the absence of an osmo-protectant. However, cells will continue to grow if an adequate stabilizer is present in the medium. In the sorbitol (osmotic protector) test, the MIC of compounds **3** and **6** increased with microbial growth, indicating interference in cellular functions that are involved the participation of the cell wall (Table 3 and Table 4).

#### 2.2.3. Association Tests—Checkerboard Method

In the microdilution assays, compound **6** demonstrated the best antifungal activity, so it was tested in combination with nystatin using the Checkerboard method to analyze its synergistic effect. When tested alone, the compound presented an MIC of 626.62 µM and nystatin presented an MIC of 8.0 µM. When tested in combination, compound **6** presented an MIC of 313.31 µM and nystatin presented an MIC of 3.2 µM, thus demonstrating an additive effect (0.5 < FIC ≤ 1); see Table 5 [79].

### 2.3. Evaluation of the Antibacterial Activity of the Derivatives

According to the results shown in Table 6, when observing the antibacterial activity of the compounds against strains of *S. aureus* (ATCC-35903), *S. epidermidis* (ATCC-12228), and *P. aeruginosa* (ATCC-25853), it was verified that of the **20** tested compounds, **9** were bioactive. 4-isopropylbenzylcinnamide (**18**) was the most potent (MIC = 458.15 µM), followed by decyl cinnamate (**9**), which for all tested strains presented an MIC = 550.96 µM. Benzyl cinnamate (**10**) was equipotent (MIC = 537.81 μM) to compound **9** against *S. aureus* ATCC-35903 and *S. epidermidis* ATCC-12228, and it was less active (1075.63 µM) against *P. aeruginosa* ATCC-25853.

#### 2.3.1. Minimum Bactericidal Concentration (MBC)

Antibacterial substances can be classified as bacteriostatic or bactericidal. This can be established by calculating the MBC/MIC ratio. A bactericidal effect is considered when the MBC/MIC ratio is ≤4, and a bacteriostatic effect is considered when the ratio is >4 [76]. In the present study, it was possible to determine that the compounds with better results against bacterial strains were also characterized as bactericidal (Table 7).

#### 2.3.2. Association Test Using the Checkerboard Method

The best microdilution test results were obtained with compound **18** (for bacterial strains). This compound was then tested in combination with amoxicillin (an antimicrobial of first choice in the treatment of infections caused by *S. aureus*) to analyze its synergistic effect using the Checkerboard method.

When tested alone, the compound presented an MIC of 128.0 µg/mL. When tested alone, amoxicillin presented an MIC of 0.015 µg/mL. When tested in combination, the compound presented an MIC of 64.0 µg/mL and amoxicillin presented an MIC of 0.0005 µg/mL, demonstrating an additive effect (0.5 < FICI ≤ 1); see Table 8 [79].

### 2.4. Molecular Docking

Potential targets identified during the target docking predictions for compounds **6** and **18** in *C. albicans* and *S. aureus*, respectively, are reported in Table 9. The table contains the UniProt accessions for all predicted potential targets, the ID given to each target, the compound corresponding to each target, a brief functional description, and a column indicating whether the protein structure was retrieved from the Protein Data Bank database or obtained via homology modeling. In total, **18** potential targets were identified for compound **6** in *C. albicans* and 10 were predicted for compound **18** in *S. aureus*. In both cases, the potential targets of the chemicals covered a wide range of functions.

As described in the Methods section, the compounds were docked into the proteins listed in Table 9. The full results of the molecular docking calculations are given as Supporting Information in Table S1, and the scores of the top scored ligand conformers by target are presented in Table 10. For caTIM10, we were unable to obtain a complete homology model, and no valid docking pose was produced for caCDC42. However, three different docking conditions were explored for caTMP1: compound **6** in the absence of dUMP and 5,10-methylene tetrahydrofolate, docking in the dUMP-binding site in the presence of the cofactor, and docking in the 5,10-methylene tetrahydrofolate cavity with dUMP present.

The docking process produced 62 complexes fulfilling the selection criterion for additional analyses (aggregated Z-score larger than 1). Overall, these calculations showed that the best scored targets of compound **6** in *C. albicans* were RPD3, HOS2, and IMA1. The best docking scores in *S. aureus* were obtained for the complexes of compound **18** with FABH, MENB, and GAR. Despite being a widely used tool for computer-aided drug discovery, molecular docking tools employ very simple scoring functions. For this reason, all 62 selected complexes were subjected to MD simulations, and estimations of their free binding energies were performed based on these simulations. The use of MD simulations to refine molecular docking results has been shown to provide better estimates of free energy in ligand–receptor complex binding [80].

**Figure 3:**
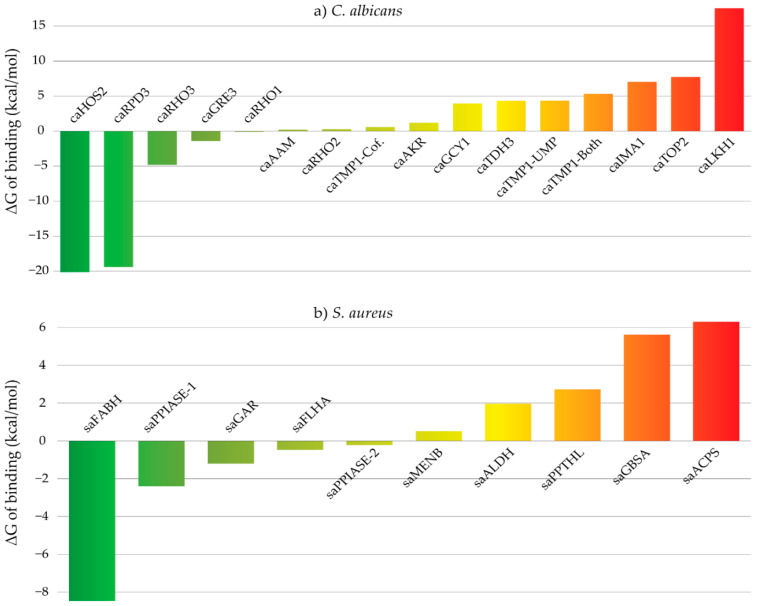
Predicted free binding energies of compounds **6** (**a**) and **18** (**b**) with their predicted targets in *C. albicans* and *S. aureus*, respectively.

Of the predicted complexes, caUGA1 and caUGA11 were excluded from the MD simulation analyses because we could not find Amber parameters for the Fe_2_S_2_ cofactor coordinated by the aspartic acid residues present in these receptors. The MD simulations led to a total simulation time of 1.14 µs for all complexes. The predicted free binding energies and their components for the **57** investigated complexes are provided as Supporting Information in Table S2. The results for the best (lowest) energy conformer by target are presented in Figure 3. The results of the free binding energy predictions agreed with those obtained from molecular docking predictions regarding the best ranked molecular target in each microorganism. That is, the most probable targets of compound **6** in *C. albicans* were caHOS2 and caRPD3, while saFABH was the most likely target of **18** in *S. aureus*. Notably, for both microbes, the free binding energies derived from the MD simulations were able to separate the top ranked targets better than molecular docking. Based on these results, the predicted binding modes for compound **6** to caHOS2 and **18** to saFABH were analyzed in detail. The 6–caRPD3 complex was excluded from the analysis because all residues in the binding cavity of this receptor and caHOS2 were conserved. In addition, the orientation of the ligand in the predicted complex with caRPD3 overlapped with that predicted for caHOS2.

**Figure 4:**
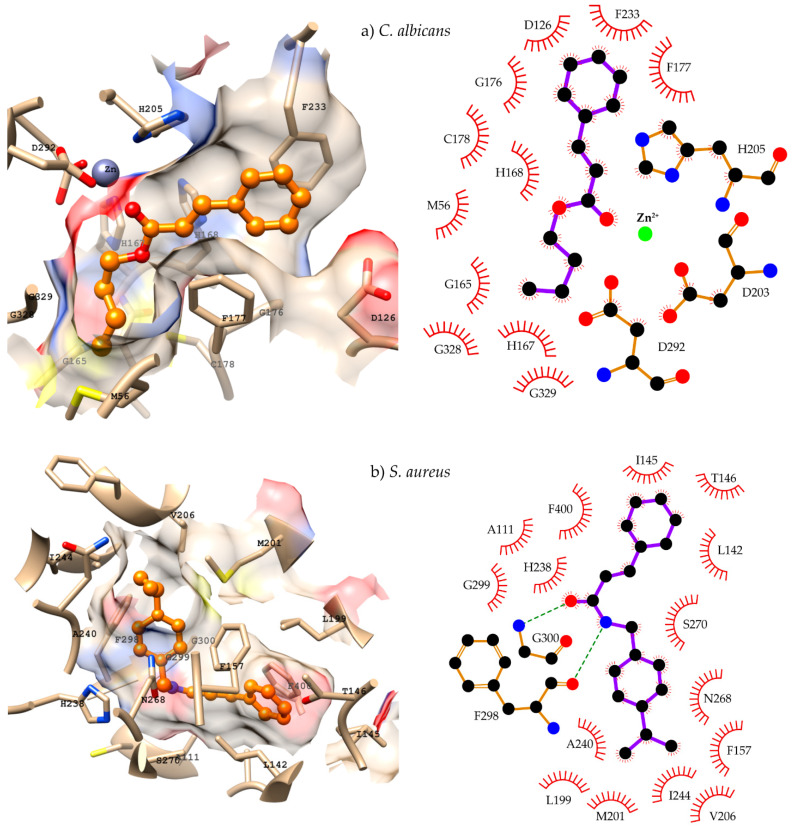
Predicted binding modes of compounds **6** and **18** to caHOS2 (**a**) and saFABH (**b**) with diagrams of the predicted ligand–receptor interactions. The ligands are represented as orange balls and sticks. Labeled residues correspond to those interacting with the receptors in at least 50% of the analyzed MD snapshots. Atoms are shown for residues either forming hydrogen bonds with the ligand or coordinating the Zn^2+^ ion.

Figure 4 shows the complexes predicted for compound **6** with caHOS2 and compound **18** with saFABH. For depiction, the 100 ligand conformations present in the same number of MD snapshots employed for MM–PBSA calculations were clustered. The centroid of the most populated cluster was selected as the representative conformation of the complex. The residues labeled in Figure 4 are those interacting with the ligands in at least 50% of the analyzed MD snapshots. The figure was prepared using UCSF Chimera [81], the frequencies of the compound–receptor interactions were analyzed with Cytoscape [82], and ligand–receptor interaction diagrams were obtained using LigPlot+ [83].

## 3. Discussion

Cinnamic acid (1) presented no activity against the studied strains. Compared with its derivative compound **2** (methyl cinnamate), it was observed that alteration of the carboxylic group to an ester function resulted in a bioactive derivative with an MIC of 789.19 μM against all tested *Candida* strains and an MIC of 1578.16 μM against *A. flavus* and *P. citrinum*.

The presence of the ethyl group in compound **3** potentiated its pharmacological response (MIC = 726.36 μM), possibly due to an increase in lipophilicity and greater penetration of the compound into biological membranes. Indeed, the increase in carbon chain length increased the antifungal action of compound **6** (butyl cinnamate, MIC = 626.62 μM). There are several studies and lipophilicity has been extensively studied and is considered one of the most important parameters influencing antifungal activity.

The compound propyl cinnamate (4) was bioactive against all tested strains, with an MIC = 672.83 μM. However, its isomer, compound **5** (R = isopropyl), was only bioactive against yeast strains (MIC = 672.83 µM).

Compounds **8** (R = isopentyl) and **7** (pentyl cinnamate) presented no activity against the tested *Candida* species. However, compound **7** was weakly bioactive against *A. flavus* and *P. citrinum,* with an MIC = 2345.39 μM. Interestingly, the higher alkyl volume at the terminal portion of compound **8** resulted in inactivity against both fungal species.

compound **9** (decyl cinnamate) was bioactive against all *Candida* strains, with an MIC = 1101.92 µM, corroborating the results of a previous study [78] that reported on decyl 4-chlorocinnamate with antifungal action against strains of *C. albicans* ATCC 90028, *C. glabrata* ATCC 90030, *C. krusei* ATCC 34125, and *C. guilliermondii* ATCC 22017 (MIC = 3.10 μmol/mL, 3.10 μmol/mL, 3.10 μmol/mL, and 1.15 μmol/mL, respectively), evidencing the contribution of the decyl group in cinnamate analogs to the antifungal action.

Reduced biological activity (MIC = 1075.63 µM and 2151.26 µM, respectively) was noticed in compound **10** (benzyl cinnamate) compared with compound **6**. The introduction of an aromatic ring as a radical, with or without substituents, resulted in a loss of antifungal activity, except for compounds **17** and **18**. The molecular volume of these groups may have influenced this change in biological potency.

Of the studied amides, only compounds 17 and 18 were bioactive. The activity of compound **17** (4-chlorobenzyl cinnamide) was weak (MIC = 2021.31 μM) and specific for filamentous fungal strains. compound **18** was bioactive against all studied fungal strains, with an MIC = 1832.62 μM for *C. albicans* (ATCC-76485) and *C. tropicalis* (ATCC-13803) strains and an MIC = 916.31 μM for *C. glabrata* (ATCC-90030), *A. flavus* (LM-171), and *P. citrinum* (ATCC-4001).

To better understand the mechanism of the antifungal action of the derivatives, compounds **4** and **6** were subjected to a test to verify the mode of action on the cell wall and plasmatic membrane of the fungus using strains of *C. albicans* ATCC-76485 and *P. citrinum* ATCC-4001. The test results (Table 3 and Table 4) showed that there was a direct interaction between the molecules and ergosterol, a component of the fungus cell membrane, and with sorbitol, an osmotic protector of the cell wall.

Antibacterial activity increased with the length of the carbonic radical chain in compound **2** (methyl cinnamate) with an MIC = 789.19 µM, in compound **3** (ethyl cinnamate) with an MIC = 726.36 µM, and in compounds **4** (propyl cinnamate), **6** (butyl cinnamate), and **9** (decyl cinnamate) with MIC = 672.83 µM, 672.83 µM, and 550.96 µM, respectively, against all studied bacteria. These results suggest an increase in the liposolubility-augmented passage through the biological membrane. compound **7** (pentyl cinnamate) with five carbons in the main chain was inactive against *S. aureus* ATCC-35903.

compound **5** (with an isopropyl group) was bioactive against all tested bacterial strains; however, compound **8** (with an isopentyl group) was inactive, confirming that a greater carbon chain length with a bulky terminal group can result in steric effects that influence bioactivity. In the aryl derivatives group, only compound **18** (4-isopropylbenzylcinnamide) was bioactive against the studied strains, with the best antibacterial profile of the entire collection (MIC = 458.15 µM). The presence of the isopropyl group attached to the aromatic ring was important for antibacterial activity of this compound. In the Checkerboard test, the compounds with the best results (**6** and **18**) both showed an additive effect when associated with the reference drug.

Subsequently, the antifungal and antibacterial activity of compounds 6 and 18 were investigated through a molecular modeling study. For caHOS2, as well as for caRPD3, the carbonyl oxygen in compound **6** directly points to the Zn^2+^ ion. Furthermore, the phenyl ring of the ligand was found to be positioned in parallel with F233 and F177 at the entrance of the binding cavity and to allow for π–π stacking with these residues. This moiety also interacts with D126. These stacking interactions, as well as the strong electrostatic interaction predicted with the metal ion at the active site, are likely to make the largest contributions towards complex stability. In addition, the central enoate group presents contacts with H167, H168, and H205. However, the butyl tail was found to occupy the bottom of the binding cavity, comprising a mostly hydrophobic tunnel flanked by M56, G165, C178, G328, and G329.

In the complex predicted for compound **18** with saFABH, the 4-isopropylbenzyl group occupies the entrance of the binding site and forms a large network of interactions that includes F157, M201, V206, H238, A240, I244, N268, and F298. Two hydrogen bonds contributing to the stability of the complex were predicted: carbonyl oxygen serves as an acceptor for the backbone of G300 and amide nitrogen acts as a donor to the F298 backbone. Finally, the other side of the compound accommodates a hydrophobic pocket formed by L119, L142 I145, T146, F157, and F400 of the second saFABH monomer. This phenyl ring orients favorable π–π stacking interactions with the latter two phenylalanine residues. These modeling results could explain both the antifungal activity of compound **6** and the antibacterial activity of compound **18**. Furthermore, caHOS2 has been shown to be a regulator of the essential Hsp90 protein [84]. In antifungal agents, HOS2 inhibition has been demonstrated against *C. albicans,* and it can even overcome resistance to azole drugs in this fungus [85]. Our results suggest that HOS2 and other histone deacetylases (such as RPD3) are potential antifungal drug targets [86,87,88]. Additionally, FABH is an essential enzyme for the synthesis of fatty acids in bacteria, and it has been investigated for its potential as a drug target [89]. In the specific case of *S. aureus*, FABH has been validated as a target of many chemical compounds presenting antibacterial activity [90,91,92,93].

Altogether, these modeling predictions were consistent with the antifungal and antibacterial activities observed for compounds **6** and **18**, respectively. Although target-specific experiments are required to test these hypotheses, the presented results will be useful in guiding future investigations of the mechanism of action of these compounds.

## 4. Conclusions

Nineteen compounds derived from cinnamic acid (**2**–**20**) were synthesized, and eight of them presented antimicrobial activity. Compounds **4** and **6** exhibited the best antifungal results, and compounds **9** and **18** presented the best antibacterial results. Compounds **6** and **18** presented an additive effect with differing antibiotics. Compounds **3** and **6** promoted antifungal activity, both acting on the cell membrane (sorbitol assay) and on the fungal cell wall (ergosterol assay). In the Checkerboard association test of the most bioactive compounds, **6** and **18** presented an additive effect towards inhibitory action. According to the computational results, the most likely targets of compound **6** in *C. albicans* were caHOS2 and caRPD3, while saFABH was the most likely target of compound **18** in *S. aureus.* For caHOS2, and for caRPD3, the carbonyl oxygen of compound **6** was found to directly point to the Zn^2+^ ion. Furthermore, the phenyl ring of the ligand was shown to be positioned parallel to F233 and F177 at the entrance of the binding cavity, thus allowing for π–π stacking with these residues. The central enoate group made contact with H167, H168, and H205, and the butyl tail occupied the bottom of the binding cavity, comprising a mostly hydrophobic tunnel flanked by M56, G165, C178, G328, and G329. In the complex predicted for compound **18** with saFABH, the 4-isopropylbenzyl group was shown to occupy the entry of the binding site and to form a large network of interactions that included F157, M201, V206, H238, A240, I244, N268, and F298. Two hydrogen bonds that contribute to the stability of the complex were predicted: carbonyl oxygen serves as an acceptor for the G300 backbone and amide nitrogen acts as a donor for the F298 backbone. compound **18** settles into a hydrophobic pocket formed by L119, L142, I145, T146, F157, and F400 of the second saFABH monomer. This phenyl ring is favorable for π–π stacking interactions with the last two phenylalanine residues. Our data indicate that these compounds can be used as prototypes to develop structural analogs with better antimicrobial profiles.

## 5. Materials and Methods

### 5.1. Chemistry

All the chemical products used during synthesis were from Sigma-Aldrich. The 400 ^1^H-NMR (nuclear magnetic resonance) and 100 MHz ^13^ C-NMR, and 500 ^1^H-NMR and 125 MHz ^13^ C-NMR spectra were recorded on VARIAN MERCURY, BRUKER-ASCEND, and VARIAN-RMN-SYSTEM spectrometers, respectively. Chemical shifts (δ) are expressed in parts per million (ppm) using TMS as an internal standard. Spin–spin multiplicities are given as *s* (singlet), *brs* (broad singlet), *d* (doublet), *t* (triplet), *q* (quartet), *qu* (quintet), *sext* (sextet), *sept* (septet), and *m* (multiplet). Column adsorption chromatography (CC) was performed on silica gel (Merck 60, 230–400 mesh); analytical TLC was performed on pre-coated silica gel plates (Merck 60 F254). Melting points were determined with a Microquímica apparatus (Microquímica equipamentos LTDA, Model MQAPF 302, Serial No.: 403/18, Palhoça, Brazil) at a temperature measurement range of 10 °C to 350 °C. All reactions were monitored with analytical thin layer chromatography.

#### 5.1.1. Synthesis of Compounds **2**–**8**

To a 100 mL flask, cinnamic acid (0.25 g, 1.69 mmol) and alcohol (50 mL) were added in the presence of sulfuric acid (0.4 mL); this mixture was then heated under reflux until the completion of the reaction (5–24 h), which was verified with single-spot TLC [51]. Spectroscopic data for the compounds in this study are available in the Supplementary Materials.

#### 5.1.2. Synthesis of compounds **9** and **14**

A mixture of cinnamic acid (0.2 g, 1.35 mmol), triethylamine (0.73 mL), and halide (1.39 mmol) in acetone (16.4 mL) was heated under reflux until a complete reaction (24 h), which was verified with single-spot TLC [51]. Spectroscopic data for the compounds in this study are available in the Supplementary Materials.

#### 5.1.3. Synthesis of Compounds **10**–**13** and **15**–**20**

A mixture of cinnamoyl chloride (0.1 g, 0.6 mmol) and the corresponding alcohol or amine (0.6 mmol) in pyridine (1.0 mL) was heated under reflux until a complete reaction (3–24 h), which was verified with single-spot TLC [94]. Spectroscopic data for the compounds in this study are available in the Supplementary Materials.

### 5.2. Antimicrobial Tests

#### 5.2.1. Microorganisms

The 20 selected compounds were checked for antifungal activity against strains of *C. albicans* (ATCC-76485), *C. tropicalis* (ATCC-13803), *C. glabrata* (ATCC-90030), *A. flavus* (LM-171), and *P. citrinum* (ATCC-4001), and their antibacterial activity was evaluated against strains of *S. aureus* (ATCC-35903), *S. epidermidis* (ATCC-12228), and *P. aeruginosa* (ATCC-25853). The strains were acquired at the MICOTECA of the Mycology Laboratory, Department of Pharmaceutical Sciences (DCF), Health Science Center (CCS) of the Federal University of Paraíba. All strains were kept on Sabouraud dextrose agar (SDA) and in brain heart infusion broth (BHI) at a temperature of 4 °C, and they were used for the assays at 24–48 h in SDA/BHIA while incubated at 35 ± 2 °C. The microorganism suspension was prepared according to the 0.5 McFarland standard to obtain 1–5 × 10^6^ CFU/mL. Standard drugs, nystatin (yeast) and amoxicillin, were used as controls.

#### 5.2.2. Determination of Minimum Inhibitory Concentration (MIC)

MIC values were determined using the broth microdilution method and 96-well U-shaped plates. In each well, 100 µL of liquid medium from Roswell Park Memorial Institute (RPMI) doubly concentrated with 100 mL of product solution was added to the first row of plate wells. Through serial dilutions, concentrations of the evaluated compounds ranging from 1000 μg/mL to 2.0 μg/mL. Subsequently, 10 µL of inoculum was added to the wells in each column of the plate and the culture medium with nystatin. The plates were then incubated at 37 °C for 24–48 h. For each strain, the MIC was defined as the lowest concentration capable of inhibiting fungal growth in the wells, visually observed in comparison with the control. All tests were performed in duplicate, and the results are expressed as an arithmetic mean of the MIC values obtained in both tests [95].

#### 5.2.3. Determination of Minimum Fungicidal Concentration (MFC)

After the MIC values were determined, 10 μL aliquots of the supernatant from the wells in which complete fungal growth inhibition (MIC, MIC × 2, and MIC × 4) was observed in the microdilution plates were added to 100 μL of RPMI broth contained in new culture plates. The plates were incubated for 24–48 h at 35 ± 2 °C. The minimum fungicidal concentration (MFC) was considered as the lowest concentration of the product that was able to inhibit the growth of microorganisms [96].

#### 5.2.4. Determination of Minimum Bactericidal Concentration (MBC)

After the MIC values were determined, 10 μL aliquots of the supernatants were removed from the wells of the microdilution plates at concentrations of MIC, MIC × 2, and MIC × 4 for each strain and inoculated into new microdilution plates containing only a BHI medium. The assay was performed in triplicate. The plates were incubated at 35 ± 2 °C for 24 h, and bacterial growth was observed. The MBC was defined as the lowest concentration capable of causing the complete inhibition of bacterial growth [96,97].

#### 5.2.5. Mechanism of Antifungal Action for Amides

(1) Sorbitol Assay: The microdilution technique was performed in the presence of sorbitol (anhydrous D-sorbitol) (INLAB laboratory) to determine the mode of action of the compounds on the cell wall of *C. albicans* CBS 562. For this test, the inoculum was prepared with sorbitol to a final concentration of 0.8 M. The plates were incubated, and readings were taken at 24 h and 48 h post-incubation. Caspofungin was used as a positive control at an initial concentration of 5 mg/mL [76,98].

(2) Ergosterol Test: This test was performed using the microdilution technique, as previously described, in the presence of exogenous ergosterol at 400 μg/mL. Nystatin was used as a positive control. The plates were incubated, and readings were taken at 24 and 48 h [76,98].

#### 5.2.6. Association Study Using the Checkerboard Method

In a 96-well plate, all the wells were filled with 100 μL of SD broth. Compounds 6 and 18 were initially dissolved in DMSO and further diluted in SD broth Then, 100 μL of the compound was added to the initial column (wells A1 to H1) and serially diluted up to the 10th column. The 11th and 12th columns, respectively, served as a drug-only control and a growth control. Similarly, a drug control was initially dissolved in DMSO and diluted in broth to obtain a 4 × concentration of the desired drug concentration. It was separately prepared in each tube for the desired concentration. One hundred microliters of the first control drug concentration was added to the first row A wells A1 to A11 but not to A12 (the growth control). This process was repeated with the respective drug concentrations for rows B to G (but not for row H). All wells were thoroughly mixed by pipette aspiration to obtain proper drug dispersion. One hundred microliters (100 μL) of the contents of each well was transferred to another 96-well plate, which was marked as a replica plate. Finally, 100 μL of the inoculum (1 × 10^3^ to 3 × 10^3^ CFU/mL) was added to all wells, and the plates were incubated at 37 °C for 7 days [79].

### 5.3. Modeling Methods

#### 5.3.1. Target Selection

The previously described homology-based target fishing approach was employed to identify potential targets for compounds 6 and 18, respectively, in *C. albicans* and *S. aureus* [74,98]. Potential targets for each compound were separately predicted using the Similarity Ensemble Approach (SEA) web server [99]. For each compound, the sequences of the targets provided by SEA were retrieved from the Uniprot database. The sequences of the targets predicted for compound **6** were then submitted to a Blast [100] search using the proteins from *C. albicans* (taxid 5476) included in the Reference Protein (refseq_protein) database using the NCBI web implementation of Blast [101]. The same process was repeated for compound **18** using proteins from *S. aureus* (taxid 1280). Two criteria were used to select the potential targets: the selected proteins had to have a shared identity of at least 45% with the proteins identified during target fishing, and at least of 75% of their sequences had to be covered by the alignment obtained from the Blast search. Proteins fulfilling the latter criteria were selected for modeling studies.

#### 5.3.2. Molecular Docking

An initial three-dimensional (3D) conformation was generated for each compound with OpenEye’s Omega [102], and partial atomic charges of the am1bcc type were added to these with MolCharge [103]. The 3D structures of a few targets were available in the Protein Data Bank (database). These targets were ACPS (PDB code 5cxd), MEMB (PDB code 2uzf), FABH (PDB code 6kvs), and GBSA (PDB code 5eyu) from *S. aureus* and TDH3 (PDB code 7u4s) from *C. albicans*. The remaining targets predicted for compounds 6 and 18 had no available 3D structure, and homology models were generated for them on the SwissModel web server [104]. Various homology models were generated for each protein, and the highest QMEANDisCo global score by potential target was selected for molecular docking calculations.

For molecular docking, the previously reported procedure was followed [74,105]. The Gold software [106] was selected for docking calculations. The binding cavities were defined from either the co-crystallized ligands or the ligands present in the templates of the homology models. Functionally relevant cofactors or metal ions were maintained in the receptor in case they were already present or were transferred to it from the model’s templates. Hydrogen atoms were added to the receptors, and residues pointing to the binding cavity were set to be flexible. The PLP scoring function was selected for primary scoring. A total of 30 docking solutions were obtained and re-scored with the ChemScore, GoldScore, and ASP GoldScore scoring functions.

Considering the average and standard deviations of the scoring values, the scores obtained with each of the four scoring functions were converted to Z-scores at a scoring function level. The resulting Z-scores were then averaged. The ligand poses with aggregated Z-scores ˃ 1 were selected for additional studies. These selected potential complexes were further studied with molecular dynamics (MD) simulations, and free binding energies were predicted from the MD snapshots as described below.

#### 5.3.3. Molecular Dynamics Simulations and Free Energy Calculations

MD simulations were carried out with Amber 20 [107] according to previously described methods [1,76]. The same protocol was applied to model all systems, parameterizing the ligands and amino acids with the gaff2 and ff19SB force fields, respectively. For cofactors, Amber parameters were obtained from the database provided by the Bryce Group at The University of Manchester: http://amber.manchester.ac.uk/index.html (accessed on 26 May 2022). Cofactors not included in the latter database such as dUMP and 5,10-methylene tetrahydrofolate were parameterized using the same approach followed for ligands. Parameters for the Zn^2+^ ion, as well as for its coordinating residues, were retrieved from the Yuan-Ping Pang lab web page: https://www.mayo.edu/research/labs/computer-aided-molecular-design/projects/zinc-protein-simulations-using-cationic-dummy-atom-cada-approach (accessed on 26 May 2022) [108].

Truncated octahedron boxes were generated to enclose the systems that were solvated with OPC water molecules. Systems were neutralized with the addition of Na^+^ and Cl^−^ ions at a concentration of 0.15 M [109]. Two stages of energy minimization were performed, the first with restraints applied to everything except the solvent and counter ions and the second with no restraints. The systems were next heated from 0 K to 300 K (the temperature during the 20 ps production runs). The heated systems were subsequently equilibrated at constant temperature and pressure for 100 ps. The final system for each complex was used as input for five production runs, each initialized with differing random velocities to better explore each complex’s conformational space. Each production run lasted for 4 ns, totaling 20 ns per complex.

The MM–PBSA method, as implemented in the MMPBSA.py script of Amber 20, was used to estimate the free binding energies. Calculations proceeded with 100 MD snapshots evenly extracted from five production runs. To compute the free binding energies, 20 snapshots per production run were extracted. Only snapshots in the 1 ns to 4 ns time interval were considered for MM–PBSA calculations. The ionic strength for these calculations was set at 150 mM.

## Figures and Tables

**Table 1:** MIC (µg/mL and µM) of compounds **1**–**20** against fungal strains.

Compounds	*C. albicans* ATCC-76485	*C. tropicalis* ATCC-13803	*C. glabrata* ATCC-90030	*A. flavus* LM-171	*P. citrinum* ATCC-4001
**1**	+	+	+	+	+
**2**	128/789.19	128/789.19	128/789.19	256/1578.16	256/1578.16
**3**	128/726.36	128/726.36	128/726.36	128/726.36	128/726.36
**4**	128/672.83	128/672.83	128/672.83	128/672.83	128/672.83
**5**	128/672.83	128/672.83	128/672.83	+	+
**6**	128/626.62	128/626.62	128/626.62	128/626.62	128/626.62
**7**	+	+	+	512/2345.39	512/2345.39
**8**	+	+	+	+	+
**9**	256/1101.92	256/1101.92	256/1101.92	256/1101.92	256/1101.92
**10**	256/1075.63	256/1075.63	256/1075.63	512/2151.26	512/2151.26
**11**	+	+	+	+	+
**12**	+	+	+	+	+
**13**	+	+	+	+	+
**14**	+	+	+	+	+
**15**	+	+	+	+	+
**16**	+	+	+	+	+
**17**	+	+	+	512/2021.31	512/2021.31
**18**	512/1832.62	512/1832.62	256/916.31	256/916.31	256/916.31
**19**	+	+	+	+	+
**20**	+	+	+	+	+
Nystatin	-	-	-	-	-
Control medium	-	-	-	-	-
Fungal growth control	+	+	+	+	+

(+) microorganism growth; (-) no microorganism growth.

**Table 2:** Minimum inhibitory concentration (MIC) and minimum fungicide concentration (MFC) of compounds, **3**, **4**, and **6**.

Compound	*C. albicans* (ATCC-76485)			*C. tropicalis* (ATCC-13803)			*C. glabrata* (ATCC-90030)		
	MIC (µM)	MFC (µM)	MFC/ MIC	MIC (µM)	MFC (µM)	MFC/ MIC	MIC (µM)	MFC (µM)	MFC/ MIC
**3**	726.36	1452.72	2	726.36	1452.72	2	726.36	1452.72	2
**4**	672.83	672.83	1	672.83	672.83	1	672.83	672.83	1
**6**	626.62	626.62	1	626.62	626.62	1	626.62	626.62	1
	***A. flavus* (LM-171)**			***P. citrinum* (ATCC-4001)**					
	**MIC** **(µM)**	**MFC** **(µM)**	**MFC/** **MIC**	**MIC** **(µM)**	**MFC** **(µM)**	**MFC/** **MIC**			
**3**	726.36	726.36	1	726.36	726.36	1			
**4**	672.83	1345.66	2	672.83	1345.66	2			
**6**	626.62	626.62	1	626.62	626.62	1			

**Table 3:** Effects of compounds **3** and **6** in the presence or absence of a protective osmotic (sorbitol 0.8 M), as well as in the presence or absence of ergosterol, against the strains of *C. albicans*.

*C. albicans* ATCC-76485				
Compounds	with Ergosterol	without Ergosterol	with Sorbitol	without Sorbitol
**3**	672.83 µM	2691.32 µM	672.83 µM	5382.64 µM
**6**	626.62 µM	2506.48 µM	626.62 µM	5012.96 µM
Caspofungin	-	-	0.5 µg/mL	1.0 µg/mL
Nystatin	8.05 µM	129.0 µM	-	-

**Table 4:** Effects of compounds **4** and **6** in the presence or absence of a protective osmotic (sorbitol 0.8 M), as well as in the presence or absence of ergosterol, against the strains of *P. citrinum*.

*P. citrinum* ATCC-4001				
Compounds	with Ergosterol	without Ergosterol	with Sorbitol	without Sorbitol
**4**	672.83 µM	2691.32 µM	672.83 µM	2691.32 µM
**6**	626.62 µM	5012.96 µM	626.62 µM	5012.96 µM
Caspofungin	-	-	0.5 µg/mL	1.0 µg/mL
Nystatin	8.05 µM	129.0 µM	-	-

**Table 5:** Synergistic effect observed with the Checkerboard method for compound **6** with nystatin against *C. albicans*.

Micro-Organism	Compound	MIC (µM)		FIC	FICI	Result
		Alone	Combined			
*C. albicans*	**6**	626.62	313.31	0.5	0.9	Additive
	Nystatin	8.0	3.2	0.4		

**Table 6:** MIC (µg/mL and µM) of compounds **1**–**20** against bacterial strains.

Compounds	*S. aureus* ATCC-35903	*S. epidermidis* ATCC-12228	*P. aeruginosa* ATCC-25853
**1**	128/841.05	512/3364.21	512/3364.21
**2**	128/789.19	128/789.19	128/789.19
**3**	128/726.36	128/726.36	128/726.36
**4**	128/672.83	128/672.83	128/672.83
**5**	128/672.83	128/672.83	128/672.83
**6**	128/626.62	128/626.62	128/626.62
**7**	+	128/586.34	128/586.34
**8**	+	+	+
**9**	128/550.96	128/550.96	128/550.96
**10**	128/537.81	128/537.81	256/1075.63
**11**	+	+	+
**12**	+	+	+
**13**	+	+	+
**14**	+	+	+
**15**	+	+	+
**16**	+	+	+
**17**	+	+	+
**18**	128/458.15	128/458.15	128/458.15
**19**	+	+	+
**20**	+	+	+
Amoxicillin	-	-	-
Control medium	-	-	-
Bacterial growth control	+	+	+

(+) microorganism growth; (-) no microorganism growth.

**Table 7:** Minimum inhibitory concentration (MIC) and minimum bactericidal concentration (MBC) of compounds **6**, **9**, and **18**.

Compound	*S. aureus* ATCC-35903			*S. epidermidis* ATCC-12228			*P. aeruginosa* ATCC-25853		
	MIC (µM)	MFC (µM)	MFC/ MIC	MIC (µM)	MFC (µM)	MFC/ MIC	MIC (µM)	MFC (µM)	MFC/ MIC
**6**	626.62	1253.24	2	626.62	1253.24	2	626.62	1253.24	2
**9**	550.96	1101.92	2	550.96	1101.92	2	550.96	550.96	1
**18**	458.15	458.15	1	458.15	458.15	1	458.15	458.15	1

**Table 8:** Synergistic effect of compound **18** with amoxicillin against *S. aureus*.

Micro-Organism	Compound	MIC (µg/mL)		FIC	FICI	Result
		Alone	Combined			
*S. aureus*	**18**	128.0	64.0	0.5	0.53	Additive
	**Amoxicillin**	0.015	0.0005	0.033		

**Table 9:** Predicted targets of compounds **6** and **18** in *C. albicans* and *S. aureus*.

UniProt Accession	ID ^(a)^	Compound	Description ^(b)^	Source ^(c)^
*C. albicans*				
A0A1D8PLH1	caTIM10	**6**	Mitochondrial import inner membrane translocase subunit TIM10	Homology
A0A1D8PHZ5	caRHO2	**6**	Rho family GTPase	Homology
P0CY33	caCDC42	**6**	Cell division control protein 42 homolog	Homology
O42825	caRHO1	**6**	GTP-binding protein RHO1	Homology
A0A1D8PH96	caRHO3	**6**	Rho family GTPase	Homology
Q5ADT3	caAKR	**6**	Aldo/keto reductase	Homology
Q5ADT4	caGCY1	**6**	Glycerol 2-dehydrogenase	Homology
P12461	caTMP1	**6**	Thymidylate synthase	Homology
Q5ADM7	caTDH3	**6**	Glyceraldehyde-3-phosphate dehydrogenase	PDB
A0A1D8PNK3	caGRE3	**6**	D-xylose reductase	Homology
Q5AHE2	caUGA11	**6**	4-aminobutyrate aminotransferase	Homology
A0A1D8PJ17	caHOS2	**6**	Histone deacetylase	Homology
A0A1D8PH55	caUGA1	**6**	4-aminobutyrate aminotransferase	Homology
A0A1D8PSA6	caRPD3	**6**	Histone deacetylase	Homology
A0A1D8PUB9	caIMA1	**6**	Oligo-1,6-glucosidase IMA1	Homology
A0A1D8PP39	caAAM	**6**	Aamy domain-containing protein	Homology
Q59NB8	caLKH1	**6**	Leucine aminopeptidase 2	Homology
A0A1D8PMM1	caTOP2	**6**	DNA topoisomerase 2	Homology
*S. aureus*				
Q5HED0	saACPS	**18**	4′-phosphopantetheinyl transferase AcpS	PDB
Q5HH38	saMENB	**18**	1,4-dihydroxy-2-naphthoyl-CoA synthase	PDB
Q7A6I1	saPPIASE-1	**18**	Peptidyl-prolyl cis-trans isomerase (PPIase)	Homology
A0A1Q8DEF0	saPPIASE-2	**18**	Peptidyl-prolyl cis-trans isomerase (PPIase)	Homology
T1YB03	saGAR	**18**	2,5-diketo-D-gluconic acid reductase	Homology
A0A8B1CIU3	saALDH	**18**	Aldo/keto reductase	Homology
A7X0K2	saFABH	**18**	3-oxoacyl-[acyl-carrier-protein] synthase III	PDB
A0A5C0VWB1	saFLHA	**18**	Alcohol dehydrogenase	Homology
Q9L4P8	saGBSA	**18**	Betaine aldehyde dehydrogenase	PDB
A0A1Q8DC81	saPPTHL	**18**	Alpha,alpha-phosphotrehalase	Homology

^(a)^ ID used for the target. ^(b)^ Description of the target. ^(c)^ Source of the protein structure: PDB from the Protein Data Bank or Homology for structures obtained from homology modeling.

**Table 10:** Docking results for compounds **6** and **18** with their probable targets in *C. albicans* and *S. aureus*.

Target	Compound	PLP ^(a)^	Z-PLP ^(b)^	GS ^(c)^	Z-GS ^(d)^	CS ^(e)^	Z-CS ^(f)^	ASP ^(g)^	Z-ASP ^(h)^	Aggregated Z-Score
*C. albicans*										
caRHO2	**6**	71.91	3.01	27.36	0.73	24.28	2.49	19.65	1.27	1.87
caRHO1	**6**	71.48	2.58	30.14	0.84	20.49	1.33	19.76	1.13	1.47
caRHO3	**6**	51.88	1.4	23.06	1.95	17.56	0.91	14.27	1.12	1.35
caAKR	**6**	47.04	1.22	12.46	1.03	21.49	1.65	29.27	1.82	1.43
caGCY1	**6**	42.72	0.18	8.85	1.26	16.08	1.31	23.79	1.33	1.02
caTMP1-Both ^(i)^	**6**	44.4	1.63	26.88	1.03	14.27	−0.73	22.16	2.39	1.08
caTMP1-UMP ^(j)^	**6**	51.26	2.06	20.96	0.32	11.16	0.65	17.58	0.37	0.85
caTMP1-Cof. ^(k)^	**6**	60.42	1.53	27.43	1.19	23.54	1.14	33.79	3.49	1.84
caTDH3	**6**	33.35	1.32	0.06	−0.1	10.49	2.38	10.67	1.42	1.26
caGRE3	**6**	44.93	1.38	19.45	1.24	13.61	1.87	19.18	1.44	1.48
caUGA11	**6**	55.78	1.77	15.07	0.77	19.41	1.13	25.73	1.99	1.42
caHOS2	**6**	66.22	1.77	26.47	0.76	25.33	2.06	29.1	1.88	1.62
caUGA1	**6**	52.47	2.31	17.77	1.13	19.68	1.78	17.4	−0.06	1.29
caRPD3	**6**	64.83	4.04	43.1	2.61	21.1	2.2	26.76	1.84	2.67
caIMA1	**6**	64.55	3.22	22.94	0.32	26.58	2.64	30.08	1.49	1.92
caAAM	**6**	40.33	1.7	−21.83	−0.15	11.56	2	18.72	2.01	1.39
caLKH1	**6**	57.37	1.77	19.55	0.29	24.73	1.69	26.95	1.3	1.26
caTOP2	**6**	40.32	1.31	8.27	1.7	12	2.02	−1.69	−0.29	1.18
*S. aureus*										
saACPS	**18**	69.08	2.87	39.36	1.29	21.73	1.23	19.88	0.46	1.46
saMENB	**18**	70.68	2.5	26.92	−0.98	29.31	2.31	26.4	0.68	1.13
saPPIASE-1	**18**	64.35	2.69	19.57	0.51	24.81	1.88	20.69	−0.21	1.22
saPPIASE-2	**18**	55.7	1.69	15.71	0.58	24.29	2.21	31.95	3	1.87
saGAR	**18**	62.95	1.58	10.82	0.51	26.03	1.5	32.66	1.29	1.22
saALDH	**18**	54.21	1.98	6.7	0.6	22.61	2.1	24.56	2.28	1.74
saFABH	**18**	74.62	2.31	14.42	0.92	34.2	1.61	35.22	2.32	1.79
saFLHA	**18**	49.49	1.37	−48.13	0.78	11.41	1.47	12.48	1.59	1.3
saGBSA	**18**	38.58	1.64	−93.97	0.26	8.62	1.47	10.47	1.57	1.23
saPPTHL	**18**	55.63	0.76	29.66	1.27	23.05	1.8	24.44	0.23	1.02

^(a)^ PLP score. ^(b)^ Scaled PLP score. ^(c)^ GoldScore score. ^(d)^ Scaled GoldScore score. ^(e)^ ChemScore score. ^(f)^ Scaled ChemScore score. ^(g)^ ASP score. ^(h)^ Scaled ASP score. ^(i)^ Docking into both dUMP and cofactor site. ^(j)^ Docking into the dUMP site. ^(k)^ Docking into the cofactor site.

## Data Availability

Not applicable.

## Supplementary Materials

The following supporting information can be downloaded at: https://www.mdpi.com/article/10.3390/molecules28041918/s1; Spectroscopic data of compounds; Table S1: Results of docking compounds 6 and 18 to their probable targets in *C. albicans* and *S.aureus*.; Table S2: Predicted free energies of binding and their components for the predicted complexes of compounds **6** and **18** with their potential targets in *C. albicans* and *S. aureus*. References [110,111,112,113,114,115,116,117,118] are cited in the Supplementary Materials.
