# Supplementary material for: Synthetic Cinnamides and Cinnamates: Antimicrobial Activity, Mechanism of Action, and In Silico Study
Source: Molecules. 2023 Feb 17;28(4):1918. doi: 10.3390/molecules28041918 (PMC9967511; doi:10.3390/molecules28041918)
Supplement: Supplementary file 1 [file molecules-28-01918-s001.zip › molecules-2204175-supplementary.pdf]

Synthetic Cinnamides and Cinnamates: Antimicrobial Activity, Mechanism of Action, and *In Silico* Study

Mayara Castro de Morais [1], Edeltrudes de Oliveira Lima [1], Yunierkis Perez-Castillo [2] and Damião Pergentino de Sousa [1,3,*]

[1] Department of Pharmaceutical Sciences, Federal University of Paraíba, João Pessoa 58051-900, PB, Brazil
[2] Bio-cheminformatics Research Group and Area de Ciencias Aplicadas, Facultad de Ingeniería y Ciencias Aplicadas, Universidad de Las Americas, Quito 170503, Ecuador
[3] Postgraduate Program in Bioactive Natural and Synthetic Products, Federal University of Paraíba, João Pessoa 58051-900, PB, Brazil
* Correspondence: damiao_desousa@yahoo.com.br

1. Experimental

1.1 Chemical characterization of compounds 2–20.

Methyl cinnamate (**2**): Amber amorphous solid; Yield 87.4% (239.3 mg, 1.47 mmol); $C_{10}H_{10}O_2$; 162.19 g/mol; m.p.: 31–33°C [1]; TLC (hexane); R_f = 0.20; IR υmax (KBr, cm^{-1}): 3063, 3030, 2946, 1710, 1639, 1579, 1450, 1309, 1176; ^{1}H-NMR ($CDCl_3$, 200 MHz): δ_H 7.65 (1H; *d*; J = 16.0 Hz), 7.46–7.45 (2H; *m*), 7.33–7.31 (3H; *m*), 6.41 (1H; *d*; J = 16.0 Hz), 3.75 (3H; *s*); ^{13}C-NMR ($CDCl_3$, 100 MHz,): δ_C 167.31 (C=O), 144.81 (C-7), 134.37 (C-1), 130.27 (C-4), 128.86 (C-3, C-5), 128.07 (C-2, C-6), 117.80 (C-8), 51.59 (C-1′) [1].
Ethyl cinnamate (**3**): Yellow oil; Yield 90.7% (269.53 mg, 1.52 mmol); $C_{11}H_{12}O_2$; 176.22 g/mol; TLC (hexane); R_f = 0.22; IR υmax (KBr, cm^{-1}): 3067, 3032, 2930, 1715, 1638, 1578, 1451, 1312, 1173, 768; ^{1}H-NMR ($CDCl_3$, 500 MHz): δ_H 7.67 (1H; *d*; J = 16.5 Hz), 7.46–7.45 (2H; *m*), 7.32–7.30 (3H; *m*), 6.42 (1H; *d*; J = 16.5 Hz), 4.23 (2H; *q*; J = 7.0 Hz), 1.34 (3H; *t*; J = 7.0 Hz); ^{13}C-NMR ($CDCl_3$, 100 MHz,): δ_C 166.83 (C=O), 144.81 (C-7), 134.43 (C-1), 130.17 (C-4), 128.82 (C-3, C-5), 128.02 (C-2, C-6), 118.24 (C-8), 60.38 (C-1′), 106 14.28 (C-2′) [1].
Propyl cinnamate (**4**): Yellow oil; Yield 90.7% (144.4 mg, 0.78 mmol); $C_{12}H_{14}O_2$; 190.24 g/mol; TLC (hexane); R_f = 0.28; IR υmax (KBr, cm^{-1}): 3064, 3032, 2969, 1714, 1639, 1579, 1450, 1313, 1174, 768; ^{1}H-NMR ($CDCl_3$, 500 MHz): δ_H 7.69 (1H; *d*; J = 16.0 Hz), 7.53–7.51 (2H; *m*), 7.38–7.37 (3H; *m*), 6.45 (1H; *d*; J = 16.0 Hz), 4.16 (2H; *t*; J = 7.0 Hz), 1.73 (2H; *sex*; J = 7.0 Hz), 0.99 (3H; *t*, J = 7.5 Hz); ^{13}C-NMR ($CDCl_3$, 125 MHz,): δ_C 167.07 (C=O), 144.58 (C-7), 134.51 (C-1), 130.22 (C-4), 128.89 (C-3, C-5), 128.07 (C-2, C-6), 118.31 (C-8), 66.14 (C-1′), 22.13 (C-2′), 10.47 (C-3′) [1, 2].
Isopropyl cinnamate (**5**): Yellow oil; Yield 83.1% (133.3 mg, 0.70 mmol); $C_{12}H_{14}O_2$; 190.24 g/mol; TLC (hexane); R_f = 0.34; IR υmax (KBr, cm^{-1}): 3063, 2981, 1710, 1639, 1579, 1450, 1309, 1176, 768; ^{1}H-NMR ($CDCl_3$, 500 MHz): δ_H 7.64 (1H; *d*; J = 16.0 Hz), 7.44–7.42 (2H; *m*), 7.30–7.28 (3H, *m*), 6.37 (1H; *d*; J = 16.0 Hz), 5.10 (1H; *septed*; J = 6.0 Hz), 1.26 (6H; *d*; J = 6.0 Hz); C-NMR ($CDCl_3$, 125 MHz,): δ_C 166.16 (C=O), 144.06 (C-7), 134.35 (C-1), 129.93 (C-4), 128.66 (C-3, C-5), 127.82 (C-2, C-6), 118.62 (C-8), 67.32 (C-1′), 21.74 (C-2′)[1–3].
Butyl cinnamate (**6**): Yellow oil; Yield 89.3% (153.8 mg, 0.75 mmol); $C_{13}H_{16}O_2$; 204.27 g/mol; TLC (hexane); R_f = 0.2; IR υmax (KBr, cm^{-1}): 3063, 3030, 2961, 1714, 1639, 1579, 1451, 1311, 1172, 768; ^{1}H-NMR ($CDCl_3$, 400 MHz): δ_H 7.69 (1H; *d*; J = 16.0 Hz), 7.53–7.51 (2H; *m*), 7.39–7.37 (3H; *m*), 6.44 (1H; *d*; J = 16.0 Hz), 4.21 (2H; *t*; J = 6.8 Hz), 1.70 (2H; *q*; J = 6.8 Hz), 1.44 (2H; J = 7.2 Hz), 0.97 (3H; *t*; J = 7.2 Hz); ^{13}C-NMR ($CDCl_3$, 100 MHz,): δ_C 167.12 (C=O), 144.57 (C-7), 134.50 (C-1), 130.21

(C-4), 128.88 (C-3, C-5), 128.06 (C-2, C-6), 118.32 (C-8), 64.45 (C-1′), 30.80 (C-2′), 19.22 (C-3′), 13.76 (C-4′)[1,3].

Pentyl cinnamate (**7**): Yellow oil; Yield 68.9% (126.9 mg, 0.58 mmol); $C_{14}H_{18}O_2$; 218.30 g/mol; TLC (hexane); R_f = 0.22; IR υmax (KBr, cm^{-1}): 3065, 3032, 2959, 1714, 1639, 1579, 1450, 1311, 1171, 767; ^{1}H-NMR ($CDCl_3$, 400 MHz): δ_H 7.68 (1H; *d*; J = 16.0 Hz), 7.52–7.50 (2H; *m*), 7.37–7.35 (3H; *m*), 6.43 (1H; *d*; J = 16.0 Hz), 4.20 (2H; *t*; J = 6.8 Hz), 1.71 (2H; *q*; J = 6.8 Hz), 1.41–1.36 (4H; *m*), 0.93 (3H; J = 7.2 Hz); ^{13}C-NMR ($CDCl_3$, 100 MHz,): δ_C 167.08 (C=O), 144.57 (C-7), 134.53 (C-1), 130.23 (C-4), 128.90 (C-3, C-5), 128.08 (C-2, C-6), 118.35 (C-8), 64.73 (C-1′), 28.49 (C-2′), 28.18 (C-3′), 22.41 (C-4′), 14.02 (C-5′) [1,4,5].

Isopentyl cinnamate (**8**): Yellow oil; Yield 76.1% (140.2 mg, 0.64 mmol); $C_{14}H_{18}O_2$; 218.30 g/mol; TLC (hexane); R_f = 0.2; IR υmax (KBr, cm^{-1}): 3064, 3034, 2960, 1714, 1639, 1579, 1451, 1311, 1168, 767; ^{1}H-NMR ($CDCl_3$, 400 MHz): δ_H.7.68 (1H; *d*; J = 16.0 Hz), 7.53–7.51 (2H; *m*), 7.38–7.36 (3H; *m*), 6.44 (1H; *d*; J = 16.0 Hz), 4.24 (2H; *t*; J = 6.8 Hz), 1.80–1.70 (1H; *m*), 1.60 (2H; *q*; J = 6.8 Hz), 0.96 (3H; *d*; J = 6.4 Hz); ^{13}C-NMR ($CDCl_3$, 100 MHz,): δ_C 167.24 (C=O), 144.62 (C-7), 134.65 (C-1), 130.22 (C-4), 129.02 (C-3, C-5), 128.16 (C-2, C-6), 118.33 (C-8), 63.32 (C-1′), 37.66 (C-2′), 25.20 (C-3′), 22.69 (C-4′)[1].

Decyl cinnamate (**9**): Yellow oil; Yield: 47.9% (105.9 mg); $C_{19}H_{28}O_2$; 288.43 g/mol; TLC (9:1 Hex:AcOEt); R_f = 0.52; IR υmax (KBr, cm^{-1}): 3051, 3030, 2932, 1703, 1640, 1593, 1440, 1310, 1187; ^{1}H-NMR ($CDCl_3$, 500 MHz): 7.68 (*d*, J = 16.0 Hz, 1H); 7.50 – 7.49 (*m*, 2H); 7.36 – 7.34 (*m*, 3H); 6.43 (*d*, J = 16.0 Hz); 4.19 (*t*, J = 7.0 Hz, 2H); 1.69 (*q*, J = 7.0 Hz, 2H); 1.42-1.37 (*m*, 2H); 1.36-1.27 (*m*, 12H), 0.88 (*t*, J= 7.0 Hz, 3H); ^{13}C-NMR ($CDCl_3$, 125 MHz,): δ_C 167.03 (C=O), 144.54 (C-7), 134.51 (C-1), 130.19 (C-4), 128.85 (C-3, C-5), 128.05 (C-2, C-6), 118.32 (C-8), 64.71 (C-1′), 31.93 (C-2′), 29.58 (C-3′), 29.35 (C-4′), 29.35 (C-5′), 29.33 (C-6′), 28.78 (C-7′), 26.02 (C-8′), 22.71 (C-9′), 14.13 (C-10′) [1].

Benzyl cinnamate (**10**): Yellow oil; Yield 52.9% (85.1 mg, 0.36 mmol); $C_{16}H_{14}O_2$; 238.29 g/mol; TLC (hexane); R_f = 0.3; IR υmax (KBr, cm^{-1}): 3066, 3032, 2921, 1717, 1637, 1578, 1450, 1309, 1163, 767; ^{1}H-NMR ($CDCl_3$, 400 MHz): δ_H 7.75 (*d*, *J* = 16.0 Hz, 1H); 7.54 – 7.53 (*m*, 2H); 7.43 – 7.38 (*m*, 8H, H-3, H-4, H-5, H-2′, H-3′, H-4′, H-5′, H-6′); 6.51 (*d*, *J* = 16.0 Hz, 1H); 5.27 (*s*, 2H); ^{13}C-NMR ($CDCl_3$, 100 MHz,): δ_C 166.03 (C=O), 145.06 (C-7), 135.28 (C-1′), 132.74 (C-1), 130.60 (C-4), 129.97 (C-3, C-5), 128.94 (C-2, C-6), 128.49 (C-3′, C-5′), 128.44 (C-2′, C-4′, C6′), 117.72 (C-8), 64.83 (C-7′) [1,4–6].

4-Methylbenzyl cinnamate (**11**): White solid; Yield 63.8% (108.6 mg, 0.43 mmol); m.p.: 40–44°C [1]; $C_{17}H_{16}O_2$; 252.30 g/mol; TLC (9:1 hexane/EtOAc); R_f = 0.52; IR υmax (KBr, cm^{-1}): 3051, 3027, 2922, 1704, 1640, 1574, 1448, 1310, 1187, 803, 768; ^{1}H-NMR ($CDCl_3$, 400 MHz): δ_H 7.76 (*d*, J = 16.0 Hz, 1H); 7.54 – 7.51 (*m*, 2H); 7.39 – 7.38 (*m*, 3H), 7.33 (*d*, J = 8.0 Hz, 2H), 7.21 (*d*, J = 8.0 Hz, 2H), 6.48 (*d*, J = 16.0 Hz, 1H), 5.32 (*s*, 2H), 2.38 (*s*, 3H), ^{13}C-NMR ($CDCl_3$, 100 MHz,): δ_C 166.10 (C=O), 145.22 (C-7), 138.54 (C-4′), 134.24 (C-1), 133.46 (C-1′), 130.40 (C-4), 129.66 (C-3′, C-5′), 129.02 (C-3, C-5), 128.61 (C-2′, C-6′), 128.26 (C-2, C-6), 118.10 (C-8), 66.47 (C-7′), 21.75 (C-8′) [1,2,5].

4-Hydroxybenzyl cinnamate (**12**): Yellow oil; Yield 70.0% (110.6 mg); $C_{16}H_{14}O_3$; 218.30 g/mol; TLC (7:3 hexane/EtOAc); R_f = 0.2; IR υmax (KBr, cm^{-1}): 3064, 3028, 2970, 1683, 1632, 1552, 1465, 1326; ^{1}H-NMR ($CDCl_3$, 400 MHz): δ_H 7.80 (1H; *d*; J = 16.0 Hz), 7.74–7.72 (2H; *m*), 7.67–7.63 (3H; *m*), 7.45 (2H; *d*; J = 8.0 Hz), 7.36-7.35 (2H; *m*), 6.65 (1H; *d*; J = 16.0 Hz), 5.19 (2H; *s*); ^{13}C-NMR ($CDCl_3$, 100 MHz,): δ_C 167.35 (C=O), 156.16 (C-4′), 145.40 (C-7), 134.43 (C-1), 130.49 (C-4, C-2′, C-6′), 129.01 (C3, C-5), 128.24 (C-2, C-6), 128.09 (C-1′), 118.02 (C-8), 115.63 (C-3′, C-5′), 66.49 (C-7′) [1,7].

4-Nitrobenzyl cinnamate (**13**): White crystal solid; Yield 62.0% (118.6 mg, 0.42 mmol); m.p.: 116–117°C [2]; $C_{16}H_{13}NO_2$; 283.28 g/mol; TLC (9:1 hexane/EtOAc); R_f = 0.28; IR υmax (KBr, cm^{-1}): 3083, 3067, 2968, 1709, 1632, 1606, 1450, 1517, 1345, 1312, 1158, 859, 749; ^{1}H-NMR (DMSO-d_6, 400 MHz): δ_H 8.23-8.15 (*m*, 2H), 7.71 (1H; *s*), 7.70–7.62 (4H; *m*), 7.39–7.35 (3H; *m*), 6.68 (1H; *d*; J = 16.0 Hz), 5.32 (2H; *s*); ^{13}C-NMR (DMSO-d_6, 100 MHz,): δ_C 165.93 (C=O), 147.11 (C-4′), 145.38 (C-7), 144.05 (C-1′), 133.91 (C-1), 130.68 (C-4), 128.96 (C-3 , C-5), 128.58 (C-2, C-6)*, 128.49 (C-2′, C-6′)*, 123.64 (C-3′, C-5′), 117.45 (C-8), 64.44 (C-7′) [1,8].

* interchangeable

4-Chlorobenzyl cinnamate (**14**): White solid; Yield 43.1% (158.6 mg, 0.58 mmol); m.p.: 62–63°C [1]; $C_{16}H_{13}ClO_2$; 272.73 g/mol; TLC (9:1 hexane/EtOAc); R_f = 0.48; IR υmax (KBr, cm^{-1}): 3065, 024, 2963, 1710, 1639, 1594, 1446, 1312, 1163, 1012, 801, 770; ^{1}H-NMR ($CDCl_3$, 400 MHz): δ_H 7.74 (1H; *d*; J = 16.0 Hz), 7.53–7.51 (2H; *m*), 7.39–7.38 (3H, *m*), 7.35 (4H; *s*), 6.48 (1H; *d*; J = 16.0 Hz), 5.21 (2H; *s*); ^{13}C-NMR ($CDCl_3$, 100 MHz,): δ_C 166.78 (C=O), 145.57 (C-7), 134.72 (C-1), 134.39 (C-1′), 134.27 (C-4′), 130.57 (C-4), 129.77 (C-2′, C-6′), 129.04 (C-3′, C-5′), 128.91 (C-3, C-5), 128.26 (C-2, C-6), 117.74 (C-8), 65.62 (C-7′) [1,3,9].

Piperonyl cinnamate (**15**): White oil; Yield 82.01% (170.9 mg); $C_{17}H_{14}O_4$; 282.30 g/mol; TLC (9:1 hexane/EtOAc); R_f = 0.37; IR υmax (KBr, cm^{-1}): 3055, 3024, 2959, 1710, 1639, 1594, 1446, 1312, 1163; ^{1}H-NMR (DMSO-d_6, 400 MHz): δH 7.69-7.68 (*m*, 2H), 7.63 (*d*, J= 16.0 Hz, 1H), 7.39- 7.36 (*m*, 3H), 6,97 (*sl*, 1H); 6.90-6.86 (*m*, 2H), 6.64 (*d*, J=16.0 Hz, 1H), 5.99 (*s*, 2H), 5.08 (*s*, 2H); ^{13}C-NMR (DMSO-d_6, 100 MHz,): δ_C 166.12 (C=O), 147.37 (C-3′), 147.19 (C-4′), 144.81 (C-7), 134.02 (C-1), 130.56 (C-4), 129.86 (C-1′), 128.95 (C-2, C-6), 128.44 (C-3, C-5), 122.31 (C-6′), 113 117.96 (C-8), 109.01 (C-2′), 108.19 (C-5′), 101.12 (C-8′), 65.68 (C-7′) [1].

Benzyl cinnamide (**16**): Yellow solid; Yield 80.5% (110 mg); m.p.: 109–111°C [10]; $C_{16}H_{15}NO$; 237.30 g/mol; TLC (7:3 hexane/EtOAc); Rf = 0.42; IR υmax (KBr, cm^{-1}): 3261, 3029, 2926, 1653, 1616, 1497; ^{1}H-NMR ($CDCl_3$, 500 MHz): δH 7.75 (1H; *d*; J = 16.0 Hz), 7.54–7.50 (2H; *m*), 7.44–7.34 (8H; *m*), 6.50 (*d*, 1H, J = 16.0 Hz), 5.26 (2H; *s*), ^{13}C-NMR ($CDCl_3$, 125 MHz,): δC 166.03 (C=O), 145.04 (C-7), 133.95 (C-1′), 132.74 (C-1), 130.64 (C-3, C-5), 129.97 (C-3′, C-5′), 128.94 (C-2, C-6), 128.49 (C-4, C-4′), 128.44 (C-2′, C-6′), 117.72 (C-8), 66.83 (C-7′) [11,12].

4-Chlorobenzyl cinnamide (**17**): White solid; Yield 58.0% (94.7 mg); m.p.: 127–129°C [13]; $C_{15}H_{12}ClNO$; 283.28 g/mol; TLC (7:3 hexane/EtOAc); R_f = 0.7; IR υmax (KBr, cm^{-1}): 3259, 3083, 2921, 1655, 1621; 1489; ^{1}H-NMR ($CDCl_3$, 500 MHz): 7.48 (*d*, H-7, J=15.5 Hz, H=1), 7.31-7.29 (*m*, H=2), 7.19-7.17 (*m*, H=3), 7.13-7.11 (*m*, H=2), 7.09-7.06 (*m*, H=2), 6.26 (*d*, J=15,5 Hz, H=1), 5.95 (*s*, H=1), 4.35 (*d*, J=6.0 Hz, H=2); ^{13}C-NMR ($CDCl_3$, 125 MHz): δC 166.02 (C=O), 141.84 (C-7), 136.92 (C-1′), 134.79 (C-4′), 133.49 (C-1), 129.96 (C-2′, C-6′), 129.33 (C-3′, C-5′), 128.98 (C-3, C-5), 128.98 (C-2, C-6), 127.95 (C-4), 120.30 (C-8), 43.23 (C-7′) [9,14].

4-Isopropylbenzyl cinnamide (**18**): Yellow oil; Yield 57.3%; $C_{19}H_{21}NO$; 239.38 g/mol; TLC (7:3 hexane/EtOAc); R_f = 0.53; IR υmax (KBr, cm^{-1}): 3283, 3092, 2921, 2839, 1654, 1621, 1489. ^{1}H-NMR ($CDCl_3$, 500 MHz): δH 7.75 (*d*, J=15.0 Hz, 1H), 7.37–7.33 (*m*, 2H), 7.29–7.27(*m*, 7H), 6.65 (*d*,J=15.0 Hz, 1H), 4.97 (*sept*, J= 6.5 Hz, 1H), 4.67-4.61(*m*, 2H), 1.16 (*d*, J=7.0 Hz, 6H); ^{13}C-NMR ($CDCl_3$, 125 MHz,): 167.27 (C=O), 142.99 (C-7), 139.03 (C-4′), 135.50 (C-1, C-1′), 129.57 (C-3, C-5), 128.80 (C-2, C-6), 127.90 (C-4, C-2′, C-6′), 126.15 (C3′, C-5′), 119.07 (C-8), 46.33 (C-7′), 46.16 (C-8′), 20.45 (C-9′) [11,13].

Piperonyl cinnamide (**19**): White oil; Yield 74.7%; $C_{17}H_{15}NO_3$; 281.31 g/mol; TLC (7:3 hexane/EtOAc); R_f = 0.57; IR υmax (KBr, cm^{-1}): 3183, 2982, 2821, 1655, 1621, 1489; ^{1}H-NMR (DMSO-d_6, 400 MHz): δH 8.48 (*t*, J=7.5 Hz, H=1); 7.48-7.45 (*m*, 2H), 7.38 (*d*, J= 16.0 Hz, 1H), 7.34- 7.25 (*m*, 3H), 6.77- 6.75 (*m*, 2H), 6.68 (*dd*, J= 8.0 Hz; 2.0 Hz, 1H), 6.59 (*d*, J=16.0 Hz, 1H), 5.88 (*s*, 2H), 4.21 (*d*, J= 6.0 Hz, 2H). ^{13}C-NMR (DMSO-d_6, 100 MHz): δC 164.94 (C=O), 147.30 (C-4′), 146.15 (C-3′), 139.00 (C7), 134.92 (C-1), 133.29 (C-1′), 129.51 (C-4), 129.40 (C-2, C-6), 127.57 (C-3, C-5), 122.52 (C-6′), 121.09 (C-8), 108.11 (C-2′, C-5′), 100.87 (C-7′), 42.18 (C-8′) [15].

Dibenzyl cinnamide (**20**): White solid; Yield 81.6% (121.2 mg); m.p.: 129-130°C [16]. $C_{23}H_{25}NO_3$; 363.45 g/mol; TLC (7:3 hexane/EtOAc); R_f = 0.48; IR υmax (KBr, cm^{-1}): 3084, 2982, 1643, 1593, 1495. ^{1}H-NMR ($CDCl_3$, 400 MHz): δH 7.85 (*d*, J=15.2 Hz, 1H), 7.45-7.42 (*m*, 2H), 7.36-7.27 (*m*, 11H), 7.22-7.20 (*m*, 2H), 6.90 (*d*, H-8, J= 15.2 Hz, 1H), 4.70 (*s*, 2H), 4.59 (*s*, 2H). ^{13}C-NMR ($CDCl_3$, 400 MHz): δC 167.36 (C=O), 143.97 (C-7), 137.46 (C-1′), 136.78 (C-1′′), 135.31 (C-1), 129.97 (C-3′, C-5′), 129.81 (C-3, C-5), 129.11 (C-2, C-6), 128.74 (C-3′′, C-5′′), 128.50 (C-2′, C-6′), 129.81 (C-3′), 129.11 (C-5′), 128.74 (C-3′′, C-5′′), 127.99 (C-2′′, C-6′′), 127.84 (C-4), 126.71 (C-4′′), 127.56 (C-4′), 117.39 (C-8), 50.20 (C-7′), 48.95 (C-7′′) [15,16].

1.2. Molecular docking

Table S1. Results of docking compounds **6** and **18** to their probable targets in C. albicans and S. aureus.

Target	Pose	PLP	Z_PLP	GS	Z_GS	CS	Z_CS	ASP	Z_ASP	Aggregated Score
					C. albicans					
RHO2	1	71.91	3.01	27.36	0.73	24.28	2.49	19.65	1.27	1.87
	2	60.54	1.05	30.56	1.01	21.64	1.52	22.41	2.14	1.43
	3	62.48	1.39	26.97	0.69	21.21	1.36	18.02	0.76	1.05
RHO1	1	71.48	2.58	30.14	0.84	20.49	1.33	19.76	1.13	1.47
	2	62.76	1.19	27.13	0.47	21.06	1.59	21.56	1.64	1.22
	3	61.77	1.03	37.09	1.71	20.00	1.10	19.07	0.93	1.19
RHO3	1	51.88	1.40	23.06	1.95	17.56	0.91	14.27	1.12	1.35
	2	53.93	1.76	15.78	0.78	17.80	0.97	12.98	0.85	1.09
AKR	1	47.04	1.22	12.46	1.03	21.49	1.65	29.27	1.82	1.43
GCY1	1	42.72	0.18	8.85	1.26	16.08	1.31	23.79	1.33	1.02
TMP1-Both	1	44.40	1.63	26.88	1.03	14.27	-0.73	22.16	2.39	1.08
	2	45.95	2.26	29.16	1.25	16.60	0.51	18.18	0.29	1.07
TMP1-UMP	1	51.26	2.06	20.96	0.32	11.16	0.65	17.58	0.37	0.85
TMP1-Cof	1	60.42	1.53	27.43	1.19	23.54	1.14	33.79	3.49	1.84
	2	59.97	1.43	33.47	1.76	22.83	0.79	27.63	1.82	1.45
	3	63.83	2.30	12.84	-0.19	23.54	1.14	26.10	1.41	1.16
	4	62.90	2.09	27.62	1.21	20.93	-0.14	24.45	0.96	1.03
TDH3	1	33.35	1.32	0.06	-0.10	10.49	2.38	10.67	1.42	1.26
	2	34.33	1.7	14.42	1.09	8.61	1.26	9.57	0.95	1.25
GRE3	1	44.93	1.38	19.45	1.24	13.61	1.87	19.18	1.44	1.48
	2	46.15	1.62	6.46	0.55	15.48	2.33	16.99	0.88	1.35
	3	43.77	1.15	9.34	0.7	12.53	1.61	17.85	1.1	1.14
UGA11	1	55.78	1.77	15.07	0.77	19.41	1.13	25.73	1.99	1.42
	2	56.53	1.93	3.48	0.04	21.09	1.62	25.15	1.84	1.36
	3	55.75	1.77	9.21	0.4	22.22	1.95	22.85	1.23	1.34
HOS2	1	66.22	1.77	26.47	0.76	25.33	2.06	29.1	1.88	1.62
	2	62.88	0.99	32.51	1.23	24.71	1.83	27.15	0.97	1.25
UGA1	1	52.47	2.31	17.77	1.13	19.68	1.78	17.4	-0.06	1.29
	2	46.35	0.58	18.39	1.21	19.23	1.62	21.6	1.06	1.12
RPD3	1	64.83	4.04	43.1	2.61	21.1	2.2	26.76	1.84	2.67
	2	52.64	1.1	19.64	0.55	18.38	0.34	27.93	2.4	1.1
IMA1	1	64.55	3.22	22.94	0.32	26.58	2.64	30.08	1.49	1.92
	2	58.95	2.15	36.19	1.95	23.99	1.75	28.42	1.11	1.74
	3	54.35	1.27	26.31	0.73	23.93	1.73	29.55	1.37	1.28
	4	54.72	1.35	32.38	1.48	23.06	1.43	26.67	0.7	1.24
AAM	1	40.33	1.7	-21.83	-0.15	11.56	2	18.72	2.01	1.39
	2	38.15	1.25	-0.16	0.81	10.8	1.82	13.74	1.08	1.24
	3	41.75	2	-19.48	-0.04	9.78	1.57	12.88	0.91	1.11
LKH1	1	57.37	1.77	19.55	0.29	24.73	1.69	26.95	1.3	1.26
	2	57.35	1.76	32.51	1.31	21.21	0.61	24.87	0.75	1.11

TOP2	1	40.32	1.31	8.27	1.70	12.00	2.02	-1.69	-0.29	1.18
	2	44.52	2.14	6.22	1.63	6.09	0.70	-0.41	0.05	1.13
	3	37.77	0.80	13.29	1.85	11.27	1.86	-2.28	-0.45	1.02
S. aureus										
ACPS	1	69.08	2.87	39.36	1.29	21.73	1.23	19.88	0.46	1.46
	2	56.51	0.26	44.63	1.66	24.74	2.34	19.82	0.43	1.17
MENB	1	70.68	2.50	26.92	-0.98	29.31	2.31	26.40	0.68	1.13
	2	67.27	1.11	44.13	1.62	27.25	1.12	25.82	0.36	1.05
PPIASE-1	1	64.35	2.69	19.57	0.51	24.81	1.88	20.69	-0.21	1.22
	2	63.94	2.62	0.23	-1.63	23.63	1.41	26.54	1.67	1.02
PPIASE-2	1	55.70	1.69	15.71	0.58	24.29	2.21	31.95	3.00	1.87
GAR	1	62.95	1.58	10.82	0.51	26.03	1.50	32.66	1.29	1.22
	2	60.20	1.11	14.32	0.69	25.19	1.17	32.31	1.22	1.05
ALDH	1	54.21	1.98	6.70	0.60	22.61	2.10	24.56	2.28	1.74
	2	50.15	1.11	14.31	1.13	21.44	1.69	20.24	0.93	1.21
FABH	1	74.62	2.31	14.42	0.92	34.20	1.61	35.22	2.32	1.79
	2	68.12	0.88	12.70	0.83	33.74	1.42	31.88	1.09	1.06
FLHA	1	49.49	1.37	-48.13	0.78	11.41	1.47	12.48	1.59	1.30
	2	48.68	1.24	-33.92	1.03	15.66	2.18	5.46	0.43	1.22
	3	51.48	1.68	-72.44	0.33	9.50	1.15	10.77	1.30	1.12
GBSA	1	38.58	1.64	-93.97	0.26	8.62	1.47	10.47	1.57	1.23
	2	36.54	1.28	-103.14	0.25	10.13	1.66	7.90	1.19	1.09
PPTHL	1	55.63	0.76	29.66	1.27	23.05	1.80	24.44	0.23	1.02

Table S2. Predicted free energies of binding and their components for the predicted complexes of compounds **6** and **18** with their potential targets in C. albicans and S. aureus.

Target	Conformer	MM-PBSA Component							DELTA TOTAL
		VD WAALS	EEL	EPB	ENPOLAR	EDISPER	DELTA G gas	DELTA G solv	
C. albicans									
RHO2	1	-31.48	-11.91	30.49	-22.91	36.09	-43.39	43.67	0.28
	2	-23.99	-9.86	24.64	-17.72	29.50	-33.85	36.42	2.57
	3	-33.95	-13.83	35.48	-23.94	37.34	-47.77	48.88	1.11
RHO1	1	-33.02	-12.67	32.02	-23.87	37.45	-45.69	45.60	-0.09
	2	-25.61	-7.22	22.96	-20.57	32.37	-32.83	34.77	1.94
	3	-25.39	-2.39	20.45	-20.12	32.29	-27.78	32.62	4.84
RHO3	1	-28.29	-3.37	15.52	-21.82	33.18	-31.67	26.88	-4.79
	2	-25.95	-3.25	22.13	-20.13	31.21	-29.19	33.22	4.02
AKR	1	-25.07	-6.59	19.82	-19.33	32.37	-31.66	32.86	1.20
GCY1	1	-26.92	-9.29	26.75	-21.10	34.51	-36.21	40.16	3.95
TMP1-Both	1	-31.22	-9.47	33.70	-24.08	40.29	-40.69	49.91	9.23
	2	-31.28	-14.18	35.13	-24.56	40.20	-45.47	50.78	5.31
TMP1-UMP	1	-35.38	-19.25	43.94	-25.03	40.07	-54.63	58.98	4.34
TMP1-Cof	1	-38.32	-15.10	35.89	-26.90	45.01	-53.42	54.00	0.59
	2	-36.36	-10.23	36.33	-26.68	43.28	-46.59	52.94	6.35

	3	-38.94	-12.99	42.89	-27.27	44.08	-51.92	59.70	7.78
	4	-38.20	-8.80	38.14	-26.67	43.13	-47.00	54.59	7.59
TDH3	1	-28.13	-6.80	25.80	-21.08	34.83	-34.92	39.54	4.62
	2	-24.65	-4.87	21.40	-19.08	31.54	-29.52	33.86	4.34
GRE3	1	-31.10	-1.33	16.71	-23.77	38.09	-32.43	31.03	-1.41
	2	-23.58	-1.33	13.37	-18.73	30.02	-24.90	24.66	-0.24
	3	-19.80	-2.50	15.96	-15.43	27.31	-22.29	27.83	5.54
HOS2	1	-28.42	-44.17	46.41	-25.34	38.90	-72.59	59.96	-12.63
	2	-24.47	-59.05	50.43	-25.35	38.31	-83.52	63.39	-20.14
RPD3	1	-27.51	-49.84	44.64	-25.73	39.03	-77.35	57.95	-19.40
	2	-28.47	-2.17	24.62	-21.42	35.36	-30.64	38.56	7.93
IMA1	1	-36.75	-0.81	27.98	-25.76	43.20	-37.57	45.42	7.85
	2	-35.32	-10.28	35.49	-25.85	43.00	-45.60	52.65	7.05
	3	-34.14	-10.14	49.52	-25.96	43.30	-44.28	66.85	22.57
	4	-35.28	-5.90	36.01	-25.52	44.06	-41.19	54.55	13.36
AAM	1	-34.27	-8.21	36.07	-25.56	42.28	-42.48	52.78	10.30
	2	-35.55	-11.79	34.91	-26.16	44.24	-47.35	52.99	5.64
	3	-35.62	-1.34	17.78	-20.03	39.41	-36.96	37.16	0.21
LKH1	1	-26.71	-14.98	41.84	-21.73	39.12	-41.69	59.23	17.54
	2	-31.51	-16.64	52.42	-23.20	41.41	-48.16	70.62	22.47
TOP2	1	-40.23	-8.04	39.31	-26.89	45.05	-48.27	57.46	9.20
	2	-40.19	-18.08	47.59	-27.02	45.44	-58.27	66.01	7.74
	3	-40.95	-20.53	55.16	-27.44	46.51	-61.47	74.22	12.75
S. aureus									
ACPS	1	-24.99	-10.76	28.02	-20.00	35.46	-35.75	43.48	7.73
	2	-24.50	-10.28	27.44	-19.77	33.41	-34.78	41.07	6.30
MENB	1	-43.29	-14.01	38.41	-31.60	54.07	-57.30	60.88	3.58
	2	-43.11	-20.70	41.81	-31.55	54.07	-63.81	64.32	0.52
PPIASE-1	1	-26.19	-9.17	20.30	-21.16	33.81	-35.36	32.96	-2.40
	2	-21.40	-4.08	14.81	-17.96	29.42	-25.48	26.26	0.78
PPIASE-2	1	-24.28	-9.12	19.62	-18.89	32.46	-33.40	33.18	-0.22
GAR	1	-30.17	-19.02	34.17	-23.82	37.65	-49.20	48.00	-1.20
	2	-25.07	-11.21	25.91	-19.74	34.69	-36.28	40.86	4.58
ALDH	1	-23.47	-13.43	26.75	-18.28	30.40	-36.89	38.86	1.97
	2	-17.78	-7.19	19.01	-14.26	24.46	-24.97	29.20	4.23
FABH	1	-48.12	-14.31	31.94	-33.66	55.69	-62.43	53.96	-8.47
	2	-45.35	-18.55	38.89	-33.27	56.01	-63.89	61.63	-2.27
FLHA	1	-45.75	-25.67	50.10	-31.67	52.52	-71.42	70.95	-0.47
	2	-32.57	-11.21	28.85	-23.93	41.91	-43.78	46.83	3.05
	3	-40.54	-8.35	32.99	-29.33	49.33	-48.89	52.99	4.11
GBSA	1	-35.40	-16.34	38.51	-27.05	45.89	-51.73	57.35	5.62
	2	-29.89	-3.12	29.44	-21.69	40.98	-33.01	48.72	15.71
PPTHL	1	-36.61	-16.75	39.38	-28.21	44.92	-53.36	56.09	2.73

References

1. Araújo, M. O., Pérez-Castillo, Y., Oliveira, L. H. G., Nunes, F. C. & Sousa, D. P. d. Larvicidal Activity of Cinnamic Acid Derivatives: Investigating Alternative Products for Aedes aegypti L. Control. *Molecule,* **2021**, *26,* 61.
2. Iranpoor, N., Firouzabadi, H., Riazi, A. & Pedrood, K. Regioselective hydrocarbonylation of phenylacetylene to α,β-unsaturated esters and thioesters with Fe(CO)5 and Mo(CO)6. *J. Organomet. Chem.* **2016**, *822*, 67–73.
3. Lutjen, A. B., Quirk, M. A., Barbera, A. M. & Kolonko, E. M. Synthesis of (E)-cinnamyl ester derivatives via a greener Steglich esterification. *Bioorg. Med. Chem.* **2018**, *26*, 5291–5298.
4. Lima, T. C.; Ferreira, A. R.; Silva, D. F.; Lima, E. O.; de Sousa, D. P. Antifungal Activity of Cinnamic Acid and Benzoic Acid Esters against Candida Albicans Strains. **2017**, *32*, 572–575.
5. Jakovetić, S. *et al.* Synthesis of Aliphatic Esters of Cinnamic Acid as Potential Lipophilic Antioxidants Catalyzed by Lipase B from Candida antarctica. *Appl. Biochem. Biotechnol.* **2013**, *170*, 1560–1573.
6. Sova, M. . *et al.* Flavonoids and cinnamic acid esters as inhibitors of fungal 17β-hydroxysteroid dehydrogenase: A synthesis, QSAR and modelling study. *Bioorg. Med. Chem.* **2006**, *14*, 7404–7418.
7. Sova, M. Antioxidant and antimicrobial activities of cinnamic acid derivatives. *Mini Rev. Med. Chem.* **2012**, *12*, 749–767.
8. Bisogno, F. *et al.* Structure-antifungal activity relationship of cinnamic acid derivatives. *J. Agric. Food Chem.* **2007**, *55*, 10635–10640.
9. Silva, R. H. N. *et al.* Antimicrobial Activity of 4-Chlorocinnamic Acid Derivatives. *Biomed. Res. Int.* **2019**, *2019*, 3941242.
10. Saito, Y., Ouchi, H. & Takahata, H. Carboxamidation of carboxylic acids with 1-tert-butoxy-2-tert-butoxycarbonyl-1,2-dihydroisoquinoline (BBDI) without bases. *Tetrahedron.* ***2008***, *64*, 11129–11135.
11. Allen, C. L., Chhatwal, A. R. & Williams, J. M. J. Direct amide formation from unactivated carboxylic acids and amines. *Chem. Commun.* **2011**, *48*, 666–668.
12. Martínez-Soriano, P. A. *et al.* Solvent-Free Synthesis of Carboxylic Acids and Amide Analogs of CAPE (Caffeic Acid Phenethyl Ester) under Infrared Irradiation Conditions. **2015**, *5*, 81–91.
13. Barajas, J. G. H., Méndez, L. Y. V., Kouznetsov, V. V. & Stashenko, E. E. Efficient synthesis of new N-benzyl- or N-(2-furylmethyl)cinnamamides promoted by the 'green' catalyst boric acid, and their spectral analysis. *Synthesis* **2008**, *3*, 0377–0382.
14. Perez-Castillo, Y. *et al.* Antifungal activity of n-(4-halobenzyl)amides against candida spp. and molecular modeling studies. *Int. J. Mol. Sci.* **2022**, *23*, 419.
15. Khaldoun, K. *et al.* An Efficient Solvent-Free Microwave-Assisted Synthesis of Cinnamamides by Amidation Reaction Using Phenylboronic Acid/Lewis Base Co-catalytic System. *Synth. J. Synth. Org. Chem.* **2019**, *51*, 3891–3900.
16. Yasui, Y., Tsuchida, S., Miyabe, H. & Takemoto, Y. One-pot amidation of olefins through Pd-catalyzed coupling of alkylboranes and carbamoyl chlorides. *J. Org. Chem.* **2007**, *72*, 5898–5900.
